# Determination of Villous Rigidity in the Distal Ileum of the Possum (*Trichosurus vulpecula*)

**DOI:** 10.1371/journal.pone.0100140

**Published:** 2014-06-23

**Authors:** Yuen Feung Lim, Roger G. Lentle, Patrick W. M. Janssen, Martin A. K. Williams, Clément de Loubens, Bradley W. Mansel, Paul Chambers

**Affiliations:** 1 Institute of Food, Nutrition and Human Health, Massey University, Palmerston North, New Zealand; 2 Institute of Fundamental Sciences, Massey University, Palmerston North, New Zealand; 3 Riddet Institute, Massey University, Palmerston North, New Zealand; 4 MacDiarmid Institute of Advanced Materials and Nanotechnology, Wellington, New Zealand; 5 Institute of Veterinary, Animal and Biomedical Sciences, Massey University, Palmerston North, New Zealand; National Institute of Agronomic Research, France

## Abstract

We investigated the passive mechanical properties of villi in *ex vivo* preparations of sections of the wall of the distal ileum from the brushtail possum (*Trichosurus vulpecula*) by using a flow cell to impose physiological and supra-physiological levels of shear stress on the tips of villi. We directly determined the stress applied from the magnitude of the local velocities in the stress inducing flow and additionally mapped the patterns of flow around isolated villi by tracking the trajectories of introduced 3 µm microbeads with bright field micro particle image velocimetry (mPIV). Ileal villi were relatively rigid along their entire length (mean 550 µm), and exhibited no noticeable bending even at flow rates that exceeded calculated normal physiological shear stress (>0.5 mPa). However, movement of villus tips indicated that the whole rigid structure of a villus could pivot about the base, likely from laxity at the point of union of the villous shaft with the underlying mucosa. Flow moved upward toward the tip on the upper portions of isolated villi on the surface facing the flow and downward toward the base on the downstream surface. The fluid in sites at distances greater than 150 µm below the villous tips was virtually stagnant indicating that significant convective mixing in the lower intervillous spaces was unlikely. Together the findings indicate that mixing and absorption is likely to be confined to the tips of villi under conditions where the villi and intestinal wall are immobile and is unlikely to be greatly augmented by passive bending of the shafts of villi.

## Introduction

While villi have long been hypothesised to augment the absorption of nutrients by increasing the surface area of the small intestinal mucosa, the mass transfer of nutrients is also dependent upon the attendant fluid dynamics [1]. In the absence of villous motility, there may be stasis of fluid in the intervillous spaces [2], [3] and absorption in the lower regions more dependent on diffusion than convection [4], [5].

Recent experimental evidence has shown that arrays of short-lived radially disposed mucosal microfolds are formed during static pendular contractions [6]. This action causes the tips of the attached villi to incline towards each other in the concavities and to diverge over the apices of microfolds. Further, fluid mechanical simulations show that these actions cause fluid to be alternately expressed from and drawn into the intervillous spaces from alternate crowding and separation of villous tips, augmenting peripheral mixing and dispersion of luminal contents [6]. This mechanism is more parsimonious than one that requires coordinated endogenous contraction of groups of villi [7], [8], [9], [10] to induce such movement of fluid.

However, the efficiency with which peripheral lumen contents could be absorbed by crowding of the villous tips depends on the relative rigidity of the villous shaft. A high degree of flexibility in the shafts of villi would allow movement of their bases to occur independently of their tips. Conversely, a degree of laxity at the point of union of the base of the villus with the adjacent mucosa would allow villous shafts to pivot with respect to the plane of the underlying mucosa and thus to accentuate crowding within the concavity between adjacent mucosal folds. Given the turgor of the interstitial tissue within the shafts of villi [11] that results from a combination of hydrostatic distension by absorbed fluids and opposing circumferential tension of the collagen fibres of the villous lamina propria [12], it seems unlikely that villous shafts would possess a high degree of flexibility. However, given the differences in the dispositions and densities of collagen fibres in the subepithelial network of the villous lamina propria from that in the pericryptal lamina propria [12], the junction between the two sites would likely allow a degree of pivoting. However to date, no work has been carried out to determine the flexibility of the villous shafts or the laxity of their bases during lumen flow.

The purpose of the current work was to determine the passive mechanical properties of villi in the absence of any villous contractile activity using microbeads to allow flow velocities and the imposed stresses to be determined directly. We also endeavour to describe the flow patterns around villi in *ex vivo* tissue in a flow cell that was designed to replicate physiological and supra-physiological levels of shear stress acting on small intestinal villi. The rigidity of villi about their longitudinal axes during flow was assessed from a sequence of high definition image sequences. Micro particle image velocimetry (mPIV) was used to delineate fluid flow at a micro level and to derive velocity fields [13], [14] at various locations around villi. Further, spatiotemporal mapping techniques developed by our research group [15], [16], [17] were used to search for any intrinsic contractile movement within the shafts of villi.

## Methods

### Preparation of intestinal samples

This study was carried out in strict accordance with the ‘New Zealand Code of Practice for the Care and Use of Animals for Scientific Purposes’. The brushtail possums are regarded as a pest in New Zealand and are widely killed by trapping and/or shooting. In this case the animals were caught humanely as part of a routine pest control operation in an approved box trap in areas of pasture in the Manawatu region of New Zealand. Permission to conduct a routine pest control operation of the brushtail possum in the stated regions was not required.

The possums were subsequently transported in the box trap to the Veterinary hospital in the Veterinary faculty of Massey University where they were with anaesthetized by a registered vet and the ileum removed prior to euthanasia with intracardiac pentobarbitone. All of these procedures were approved by the Massey University Animal Ethics Committee (MUAEC approval no 12/77), which is accredited within New Zealand and internationally.

We used sections of the wall of the terminal ileum of the brushtail possum (*Trichosurus vulpecula*) maintained *ex vivo* in a flow cell. Villous intestinal mucosa can be maintained *ex vivo* for significant periods of time, provided it is well oxygenated and adequately supplied with nutrients [17], [18].

Seven freshly trapped brushtail possums (*Trichosurus vulpecula*), of either sex and between 2 and 3 kg body weight were each fasted for 4 hours and anaesthetized in an induction chamber with 5% halothane in 33% oxygen and 66% nitrous oxide administered via a face mask attached to a Bains circuit. The gut was accessed via a ventral midline incision and a 10 cm length of terminal ileum was excised. The segment of excised gut was opened by a lengthwise cut and immediately placed with mucosa uppermost in carboxygenated Earle's-Hepes solution (HBS) (NaCl 124.0, KCl 5.4, MgSO_4_ 0.8, NaH_2_PO_4_ 1.0, NaHCO_3_ 14.3, Hepes 10.0, CaCl_2_ 1.8, glucose 5.0 mM) maintained at 37°C. This procedure diluted any adherent digesta and allowed it to float clear of the mucosal surface.

**Figure 1:**
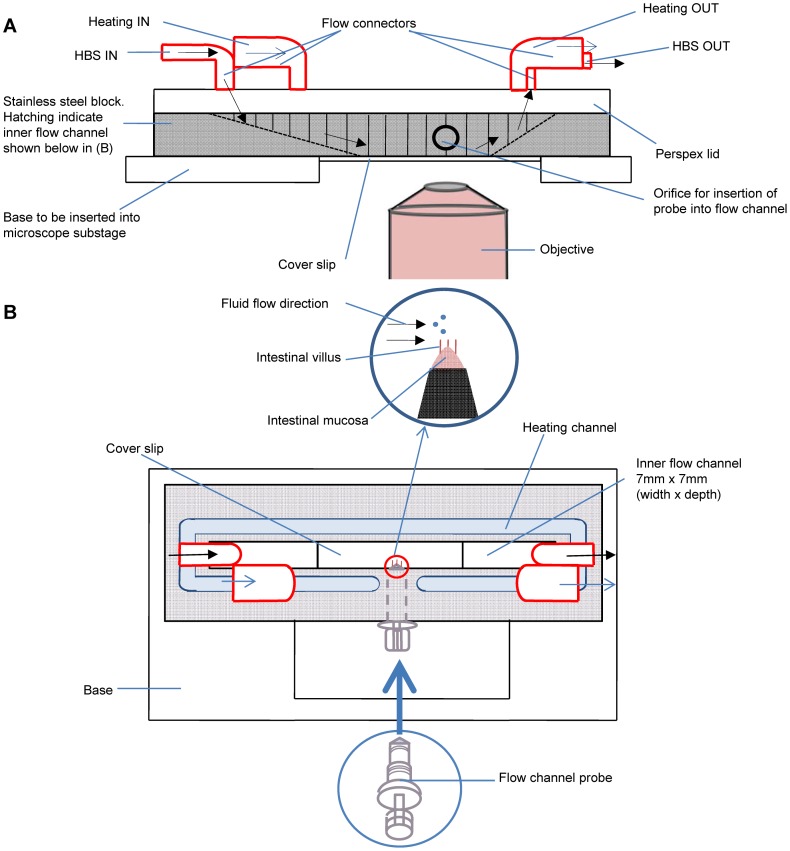
Tissue flow cell used in experimental work. A) Lateral view, B) plan view.

A 2 cm^2^ piece of mucosa was cut from the opened segment with its center located 5 cm from the distal end of the section of terminal ileum. The rectangular piece of mucosa was then draped over the 5 mm diameter conical tip of the flow cell probe (Fig. 1B) with the mucosal surface outermost, using four haemostats, one attached to each corner, and secured with a suture that was located over a grove situated just below the tip of the probe.

The tip of the probe was then extended until the overlying tissue was taut and the surplus mucosa around the suture trimmed off. The probe bearing the tautened mucosa was then mounted in a specially designed flow cell (Fig. 1) that allowed the tip and overlying mucosa to be positioned in the inner flow channel of the flow cell that was perfused with HBS. The flow cell was maintained at 37°C by the flow of an outer channel of heated water (Fig. 1B). The position of the probe was such that the tissue overlying the tip lay in the focal plane of an inverted microscope (Nikon Eclipse TE2000-U) and gave a lateral view of villi with the perfusate flowing at right angles to their long axes. Shear rates in species such as the rat and the possum during either pendular or peristaltic activity has been reported to be around 0.5–1 s^−1^ [6], [19]. Given that the viscosity in the continuous fluid layer around the tips of ileal villi of the possum is around 1.5 mPa.s [17], then by assuming Poiseuille flow we can calculate the shear stress acting on the villi at these shear rates to be between 0.7–2.5 mPa. The delivery of shear stress to villi was by perfusion of the flow cell with either HBS alone or HBS containing suspended microbeads delivered by a 50 mL syringe mounted in a syringe pump (Harvard Instruments). The syringe pump was adjusted to deliver perfusion at 3.8, 7.6, and 15.3 mL/min. The rates were chosen as they encompass reported physiological and supra-physiological levels of flow [20], [21]. Velocity profiles of the flow measured by mPIV above the villi subsequently confirmed these orders of magnitude (see Results).

The substage of the microscope on which the flow cell was mounted could be manually adjusted to view selected villi. The inverted microscope was mounted on an air damped table (Photon Control) and was equipped with a halogen bright field illuminator (Nikon), and a 10×0.3 NA (CFI Plan Fluor DL) objective lens. Image sequences for processing by micro-PIV (see below) were recorded with a Foculus FO124SC CCD camera (that was mounted on the microscope) for between 30 and 60 seconds at a frame rate of 43 Hz and a camera resolution of 640×480 pixels. In all cases, acquisition of image data commenced within 10 minutes of the time the segment was excised from the ileum. The total time elapsed following excision was determined for all image sequences.

The circulation of the heating water was halted for at least 20 seconds prior to image capture to prevent interference from flow induced vibrations. Perfusion of the inner flow channel with HBS was halted one minute prior to image capture but recommenced 5 seconds after imaging had started. The latter procedure allowed the position of the villus under static conditions to be determined. Circulation of heating water was re-established immediately on completion of recording and maintained for a minimum of 5 minutes before further recording of image sequences. All image sequences were completed within 90 minutes of the time of excision of the ileal segment.

### Micro-PIV

Polystyrene microbeads (Polysciences, Warrington, PA, USA) 3 µm in diameter were suspended at a concentration of 0.0005% (weight/volume) in the HBS perfusate. The magnitude and pattern of the two dimensional (2D) fluid flow around intestinal villi were assessed from the movement of these microbeads as measured using micro particle image velocimetry (mPIV). It is noteworthy that the method of preparation effectively removed large digesta particles and also fine food particles while mucus islands, microflora and sloughed epithelial cells [22], [23] could be distinguished from microbeads and not inadvertently tracked. Again as such fine particles were of low mass, they would move with the microbeads during bulk flow and would be unlikely to hinder their passage.

The mPIV technique used was implemented using software (PIVlab v.1.32) [24] run in the MATLAB 2011b (MathWorks, Natick, MA) programming environment. The resolution of the velocity flow fields generated by mPIV was enhanced by a technique known as ensemble correlation [25]. This technique may be applied to image sequences by two methods. Firstly, arrays of particles in a series of closely spaced focal planes could be used to determine a mean vector. Secondly, ensemble images were formed from groups of images taken of the same field of view but at different times, when the population of beads contained within them were different (the initial population of beads having left the frame by being driven by the flow perfusion). A mean vector associated with each spatial region may then be derived from the superimposed images. For example, by the latter method, for two successive groups of 100 images, the first image in each group would be superimposed, the second on the second etc. [26]. Given that the first method could not be utilized with PIVlab, the second method of enhancing the resolution of the velocity field was used. Hence the flow field was evaluated using a series of interrogation windows in which the flow vector was ultimately determined (the areas occupied by the villi were masked). The software employed a series of four interrogation windows of decreasing size that were uniformly distributed over the image in order to increase spatial resolution of the generated velocity vectors while minimizing noise. Ultimately this method was able to simultaneously determine flow velocities that varied by 2-3 orders of magnitude [26]. The interrogation window used was always 4 times the size of the maximum expected particle displacement in accordance with the one-quarter rule of mPIV for optimum recoverability of flow field velocities [14], [27]. The cross-correlation functions between successive superimposed images were evaluated by Fast-Fourier Transform (FFT) and the position of maximum in the cross-correlation was located to sub-pixel accuracy by fitting a Gaussian function to the peaks in the cross-correlation data. The velocity was determined by calculating the displacement of this maximum with respect to the prior superimposed image over a given time interval (dt = 1/43 seconds). This procedure was repeated for all interrogation areas to determine their respective velocity flow fields. The above procedure was applied to at least 100 pairs of successive superimposed images and the resulting velocity fields to be used in further analysis were obtained by averaging the velocity fields over all instances of the same time interval.

### Analysis of micro-PIV results

The magnitudes of the velocity vectors of microbeads were assessed at a number of differing volumetric flow rates, namely; at 2.0, 1.0, and 0.4 mL/min in open channel studies on the flow cell. This procedure recovered flow profiles that were close to that predicted assuming the law of Poiseuille. The same procedure was used with the tissue *in situ* to determine velocity profiles 200 µm above the tip of a chosen villus at volumetric flow rates of 38.2, 15.3, 7.6, 3.8, 1.9 mL/min. The distance of 200 µm was chosen as it was the maximum vertical distance from the villous tip that was visible in the apparatus. The mean velocity was calculated from between 20 and 30 values at perfusion flow rates between 1.9 and 7.6 mL/min, and from between 140 and 160 values for flow rates above this.

The gradation of the horizontal component of flow velocities at right angles to the long axis of the villi (U_x_) was determined at HBS perfusion rates of 15.3, 7.6, 3.8 mL/min. Plots of the horizontal component of velocity of the flow at right angles to the long axis of the villi (U_x_) were prepared from all image sequences that were suitable for the calculation of TD. Horizontal velocities were recorded at a distance of 30 µm lateral to the edge of the villous image, starting at a point 100 µm above the villous tip and at all available points below this as far down the length of the villus as was practicable (in some cases up to 400 µm below the villous tip). The starting distance of 100 µm above the villous tips was chosen as it was the greatest distance above them at which at least 15% of the mean villous length could be seen in the same visual field in all image sequences.

The gradation and flow direction of the component of flow velocity parallel to the long axis of villus (U_y_) was also determined at the same three flow rates. This was done at distances between 5–15 µm from the lateral edge of the villous image at all available points along both the upstream and downstream longitudinal axes of villi, from the tip to as far down the length as practicable (in some cases up to 400 µm below the villous tip). Only image sequences that recorded streamlines of microbeads perpendicular to the long axis of villi for the whole length of villi that could be seen in the image, which had villous lengths of over 280 µm (greater than half the mean length of a villus) were suitable for this assessment.

As the technique used for determining velocity profiles around villi involved direct superimposition of video images taken prior to the commencement of perfusion and intervals thereafter, it also allowed direct comparisons of the villus profile. This allowed us to determine whether there had been any swelling of the tissue over the course of the experiment.

### Mean villous length and width

Further samples of distal ileal mucosa, each of 2 cm^2^ in surface area, were obtained from a further four possums. Each sample was tied to the probe from the tissue flow cell and stained with 1% Bromothymol-Blue (BTB) before being mounted in a micromanipulator, and immersed in a petri dish of oxygenated HBS situated in the focal field of a Diaphot inverted microscope (Nikon). The sample was illuminated using bright field illumination from a FL-150 fibre optic lamp (MeijiTechno) and images of villi captured with a Nikon DS-Fi1 Hi-Definition digital camera. The lengths of component villi were measured along their long axis from their tips to the points where the cylindrical profiles of their shafts broadened at their junction with the basal mucosa. The width of each component villus was also determined at the midpoint of its length.

### Total displacement (TD) of the villous tip

The overall movement of the tip of a villus, i.e. total displacement (TD), was determined as the linear displacement of a point located 20 µm below its tip from its original position (when there was no flow) to that when there was steady fluid flow of HBS at each of three perfusion rates (3.8, 7.6 and 15.3 mL/min). The TD will be the sum of any linear displacement of the villous tip from bending of its shaft plus that from any pivoting around the point of attachment of its base to the mucosa, that from any translational movement of the base of the villus with respect to the adjacent muscular wall and that from any change in its lateral profile by twisting about its long axis.

**Figure 2:**
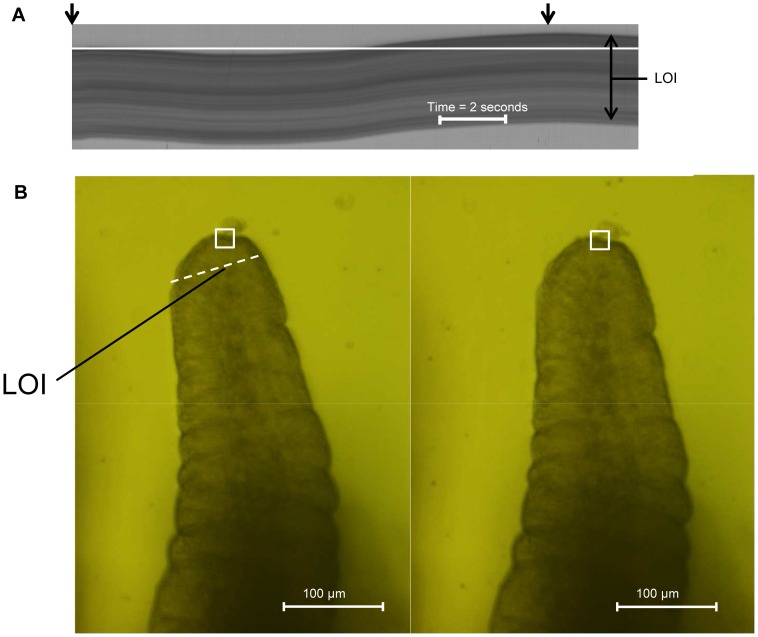
Method for determining the total displacement of villus tips (TD). A) A spatiotemporal intensity (ST) map taken along an LOI (indicated by the dashed line in (B) situated 20 µm below the villus tip before flow commenced (left arrow on upper border of map) and at the time of maximum displacement (TD) (right arrow on upper border of map). B) Views of the villus from which the ST map was taken before flow commenced and at the time of maximum displacement (right). The square marks a distinctive structural feature used as a reference point. The value of TD is calculated from the difference in the location of the same distinctive feature in the two images.

The point in time at which the maximum TD occurred following the commencement of flow was determined, from intensity based spatiotemporal mapping [16] by assessing the displacement along a line of interest (LOI) drawn at right angles to the longitudinal axis of the villus across a point 20 µm below the tip. A reference line was drawn, using Image J software (NCBI), along the temporal axis of the spatiotemporal intensity map from the position of the upstream edge of the LOI when there was no flow (Fig. 2A). The time of maximal TD could thus be identified as the point of maximum distance of the edge of the LOI from the marked line (Fig. 2B), and the requisite video image then selected. A distinctive point was then identified on the tip of the villus in one of the initial frames taken during stasis and in the image acquired at the time of maximal displacement. The linear distance between the two positions was then determined from the requisite cross-correlation data between the two images.

### Assessment of bending of the villous shaft and angular displacement

This procedure was undertaken on a series of villi using the same images that were used to determine TD and the same cross-correlation techniques applied to each in the series of selected features. The contribution of bending to the total displacement of a given villous shaft (TD) was assessed from the displacements, in the image taken at maximum displacement, of a series of features along the length of the villus that were aligned with its longitudinal axis in the initial image. Were the villus a rigid structure that could be displaced only by pivoting, (i.e. hinging about its base) the displacement distance would be expected to decrease in a linear fashion with distance from the villous tip. Conversely, were displacement to be generated by bending, then the displacement distance would vary in a non-linear fashion according to the local elasticity at the point along its length at which it was determined. Hence, the displacements of individual villi plotted against the distance from the villous tip at which they were taken, would be linear in the first case (assuming that the mechanical properties of villi did not vary along their longitudinal axes) and non-linear in the second. Likewise, if bending occurred at higher velocities of flow, the fit would depart from linearity and influence the slope of the line of best fit.

The correlation coefficient (R^2^) of the straight line regression (SLR) of the percentage displacement of the requisite TD against the distance from the villous tip was used as a basis of assessment of the quality of linear fit. Comparisons of the correlation coefficients obtained on SLRs of individual villi by ANOVA could then be used to assess whether the quality of linear fit declined significantly over the three flow rates that were used as would occur with bending.

Similarly, the difference between the slope of the line of the SLR in the plot of the displacement of the requisite TD in microns against the distance from the villous tip in microns will in each case indicate the angle with which the villus pivoted. Again comparisons by ANOVA of pivot angles of individual villi at the various speeds of flow will indicate whether pivoting continues to increase as flow increases.

### Assessment of endogenous villous motility

The occurrence of any spontaneous longitudinal movement in individual villi was assessed from spatiotemporal maps of strain rate along three lines of interest (LOI) that were orientated along longitudinal axis of villi, i.e. on the upstream and downstream edges and along the central axis. The manner in which these maps are derived has been fully described elsewhere [16]. Briefly, the software identifies displacements of textural patterns within the images of the villi along the LOI, which are plotted row by row through time. Longitudinal strain rate is then derived by differentiation with respect to distance and similarly plotted. The resulting spatiotemporal map allows regions of contraction (negative strain rate) to be distinguished from those in which extension (positive strain rate) is taking place, through time [16].

### Sampling and statistics

All statistical analyses were conducted using the SPSS statistical package version 20.0.0 (SPSS Inc., Chicago, Illinois). Normality of the various data sets generated in this work was assessed by Lillefors test. Parameter values that were not normally distributed were converted via the Johnson algorithm in Minitab or by standard mathematical manipulation. Raw non-parametric data was summarised graphically as boxplots and reported as median and interquartile range (IQR) values in the text. Converted data was compared by one-way ANOVA. The fits of displacements of various points along the shafts of villi expressed as a percentage of total tip displacement (TD) to the corresponding distance along the villus were explored by curve fitting using CurveExpert v.1.4 software [28] to determine whether the best fitting form of the regression line was linear.

## Results

### Villus length and width

Mean villous length (546±3 µm) was normally distributed while mean villous width (156±1 µm) was normally distributed only after logarithmic transformation (n = 234) in data obtained from the terminal ileum of four possums.

Direct superimposition of video images taken prior to the commencement of perfusion and intervals thereafter (see method for determination of villus bending) showed no tendency for the second image to be wider than the first i.e. no detectable change in villus width over time.

### Flow profiles around villi

**Figure 3:**
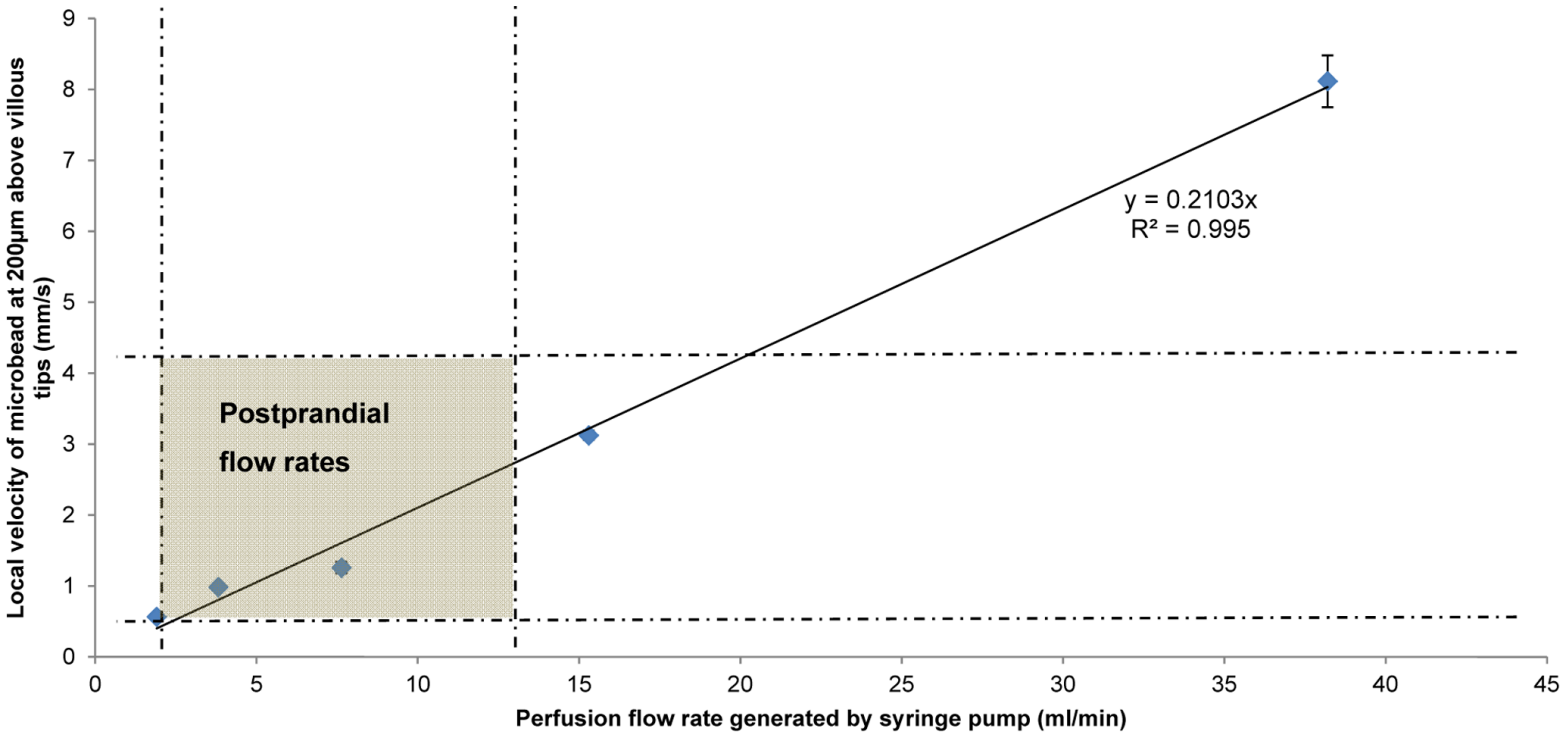
Relationship between local velocity of microbeads and the volumetric perfusion rate. The local velocity of microbeads were taken in the region 200 µm above the villous tips and determined by mPIV. Physiological flow rates reported during the postprandial period [20], [21] are indicated by the shaded region in the plot.

The relationship between perfusion rates, i.e. volumetric flow rates between 1.9 and 38.2 mL/min, and magnitude of the velocity of microbeads at a point 200 µm above the villous tips (Fig. 3) was linear as expected. This indicates that the mPIV method was sensitive enough to detect changes in flow velocity under the experimental conditions.

At all three flow rates, flow around the tips of isolated villi was characterised by streamlines moving upward toward the tip on the surface facing the flow and downward from the tip on the opposite surface. In the region located between the base of the villus and a point 50 µm below the tip, the mean velocity of the flow in the two dimensional (2D) plane examined (0.29–0.38 mm/s) was low compared to that at the tip (0.81–0.97 mm/s). These low values were likely from streamlines that had been deflected around the base of the villous.

#### Flow velocity profile perpendicular to the villous longitudinal axis

The velocity of flow at points 200 µm or more above the villi varied linearly with the distance from the tip. The approximate magnitudes of shear and strain rates acting on the villous tips could thus be calculated if it was assumed that this relationship could be extrapolated to the region at the villus tip. These shear rates varied between 1 and 7 s^−1^ and corresponded to shear stresses of 2 and 9 mPa (values that were calculated assuming Poiseuille flow in the flow cell) at perfusion rates of 3.8 and 15.3 mL/min respectively. These levels of shear stress exceeded those generated during normal peristaltic or pendular activity (see section 2.1 of Methods).

**Figure 4:**
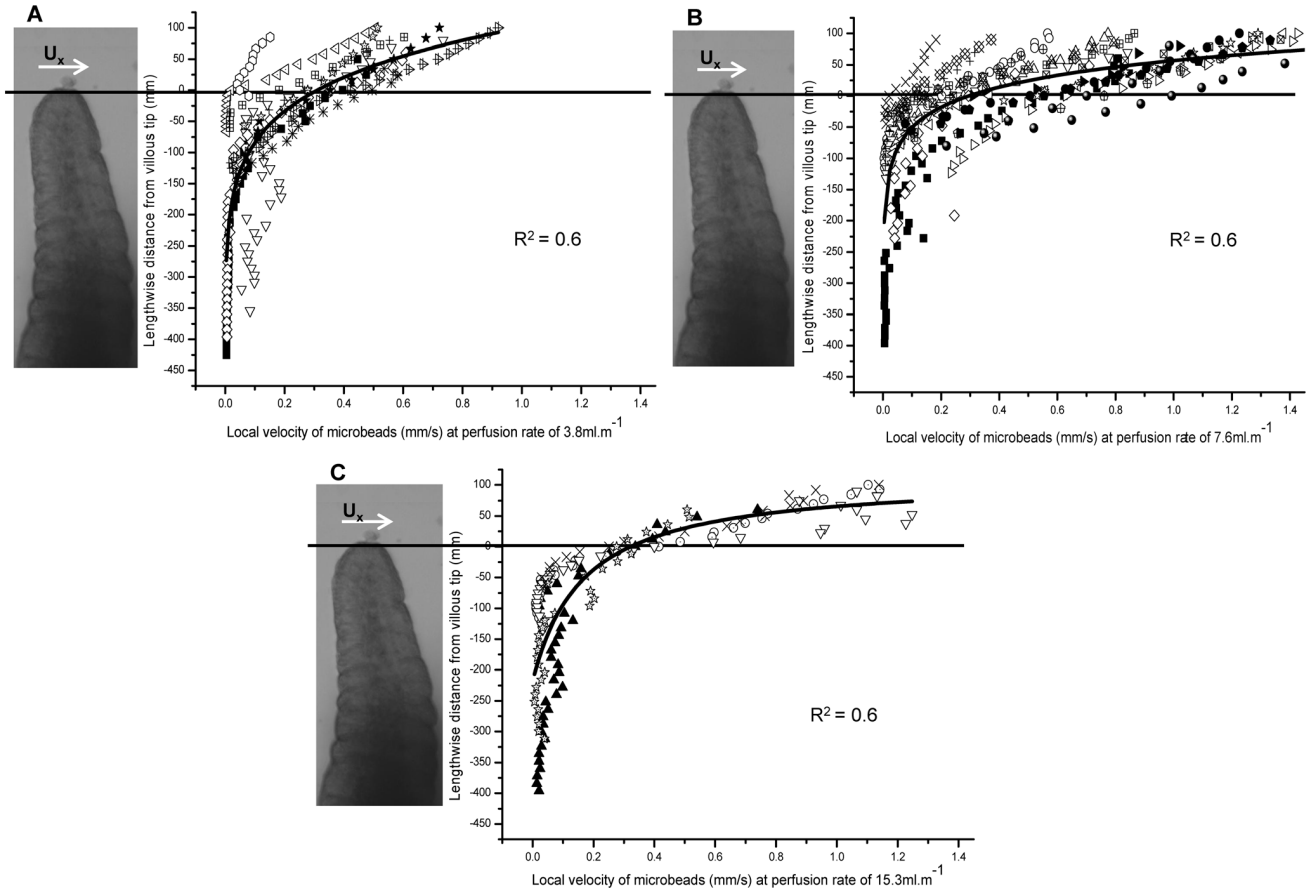
Variation of U_x_ with lengthwise distance from the villous tip. Flow velocity component at right angles to the long axis of the villi (U_x_) were determined 30 µm lateral to the villous image edge at three perfusion flow rates – A) 3.8 mL/min, B) 7.6 mL/min, C) 15.3 mL/min. Zero on both Y axes corresponds to tip of the villus while negative distances are distances below the villous tip. Exponential fits (R^2^ given on each plot) were obtained for all three perfusion rates. The different symbols on each plot represent a different experimental runs on different villi.

Plots of the horizontal component of velocity of the flow at right angles to the long axes of the villi (U_x_) showed that flow declined rapidly below the villous tips at all three flow rates (Fig. 4). The results from each experimental run (plotted as different symbols on Fig. 4) differed in absolute values but all showed a pattern of exponential decline along the length of the villus regardless of flow rate. Hence, U_x_ varied somewhat between 1.5 mm/s and 0.25 mm/s in regions above the tips of the villi according to perfusion rate and declined rapidly and exponentially below the villous tips regardless of flow rate to a mean velocity of 0.11 mm/s at approximately 100 µm below the villous tips, and less than 0.05 mm/s at 200 µm below the tips (Fig. 4).

#### Flow velocity profile parallel to the villous longitudinal axis

Of a total of 38 image sequences that were used for the calculation of TD, eight image sequences from a total of three villi from three animals were suitable (see experimental methods) for analysis of flow velocity profiles parallel to the villous longitudinal axis (U_y_) at perfusion rates of 15.3, 7.6, 3.8 mL/min.

**Figure 5:**
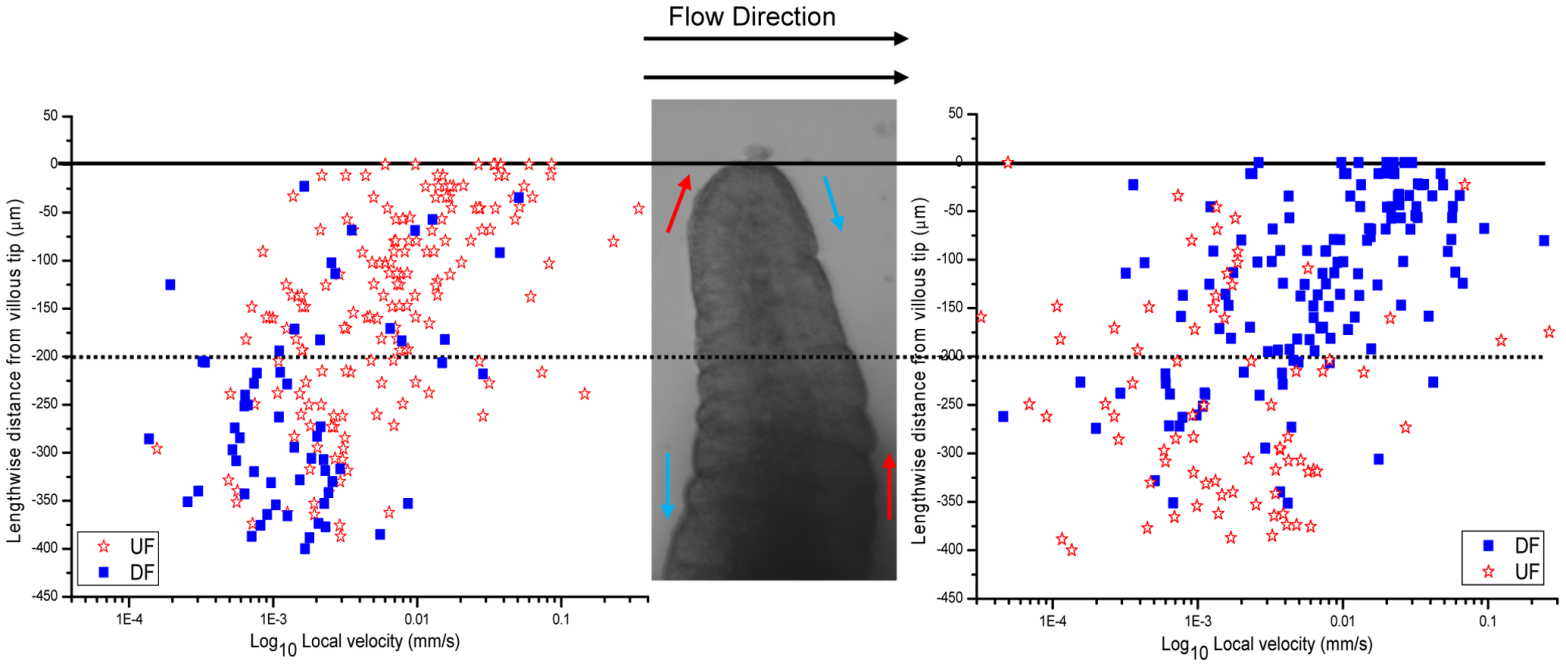
Variation of U_y_ with lengthwise distance from the villous tip. Flow velocity component parallel to the longitudinal axis of villi (U_y_) were determined 5–15 µm from the upstream edge (left) and downstream edges (right). Hollow points indicate upflow (UF) and solid points indicate downflow (DF). There is an overall trend of upward flow on the upper upstream edge and downward flow on the upper downstream edge. Local velocities are lower at distances >200 µm below the tip on both sides of the villus.

Lengthwise velocity (U_y_) tended to decrease with distance from villous tip on both the upstream and downstream edges. Hence, at distances 50 µm below the villous tips, U_y_ varied between 1 and 400 µm/s whilst in regions between 50 µm and 300 µm below the villous tips, U_y_ was more than an order of magnitude lower, varying between 0.05 and 20 µm/s (Fig. 5).

All three plots of the variation of U_y_ with lengthwise distance from the tip of the villus (Fig. 5) showed a general trend of flow towards the villous tip on the upstream edge of the villus with some flow towards the base in the lower third of villus (notably between 250 and 400 µm from the villous tip). Conversely the velocity profile plot of the downstream edge of the villus showed a general trend of flow toward the villous base. Hence, volumetric flow at physiological rates was sufficient to establish a pattern of flow around the periphery of the villus although in all cases the flow rate was low.

### Total displacement (TD) of the villous tip

**Figure 6:**
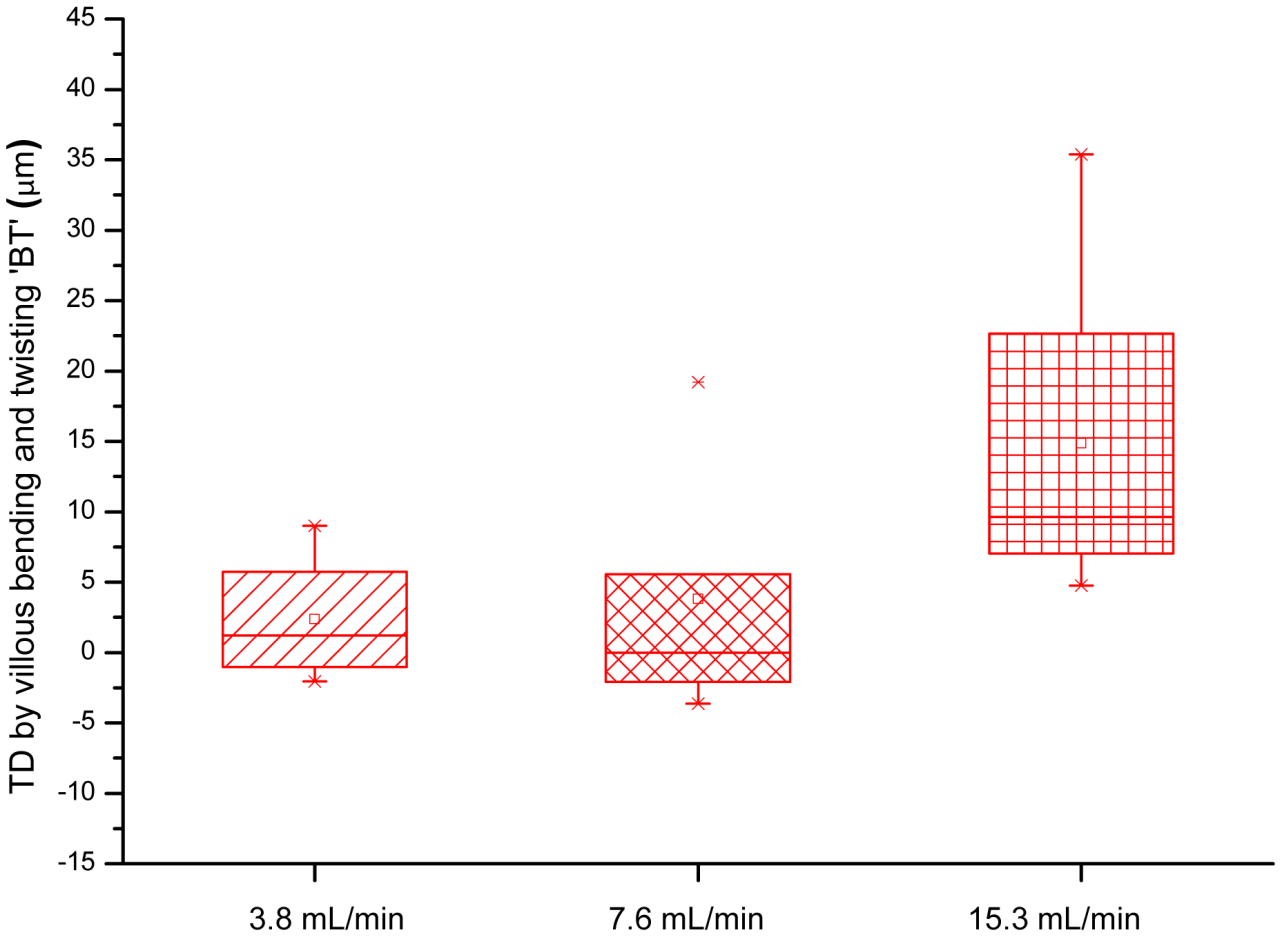
Variation of TD with volumetric flow rate. A box plot showing the variation of TD with perfusion flow rate is presented. There was no significant variation of TD with flow on ANOVA of logarithmically transformed data.

It was possible to determine TD for a total of 26 villi from 7 animals over a total of 38 image sequences (Fig. 6). The median TD was 5.07 µm with an interquartile range (IQR) of 5.29 (n = 12) at a volumetric flow rate of 3.8 mL/min; 6.46 µm with an IQR of 9.01 (n = 22) at 7.6 mL/min; and 10.59 µm with an IQR of 2.3 (n = 5) at 15.3 mL/min (Fig. 6). Thus TD was generally small, being less than 7% of the mean width of a villus. There was no significant change (P>0.05) in TD with flow rate on one-way-ANOVA of logarithmically transformed data. There was no significant variation of TD with time over a period of 80 minutes.

### Assessment of villous rigidity

The overall mean R^2^ value obtained by SLR of percentage displacement of TD against distance down the villus for all villi was 0.82±0.03. The R^2^ values obtained from individual villi (0.80 at a flow rate of 3.8±0.04 mL/min; mean 0.81±0.04 at 7.6 mL/min; 0.88±0.04 at 15.3 mL/min) did not vary significantly with flow rate. The overall mean pivot angle obtained from SLR of displacement in microns against distance down the villus in microns was 0.98^o^ with a 95% confidence interval (CI) between 0.73^o^–1.32^o^. The pivot angles of individual villi required log transformation prior to ANOVA. The pivot angles of individual villi (mean 0.65^o^; 95% CI 0.4^o^–1.06^o^ at a flow rate of 3.8±0.04 mL/min; mean 1.15^o^; 95% CI 0.76^o^–1.75^o^ at 7.6 mL/min; mean 1.27^o^; 95% CI 0.03^o^–3.76^o^ at 15.3 mL/min) also did not vary significantly with flow rate. Hence, there was no evidence of significant bending or any increase in pivot angle at any flow rate, the latter being no more than one degree in all readings.

**Figure 7:**
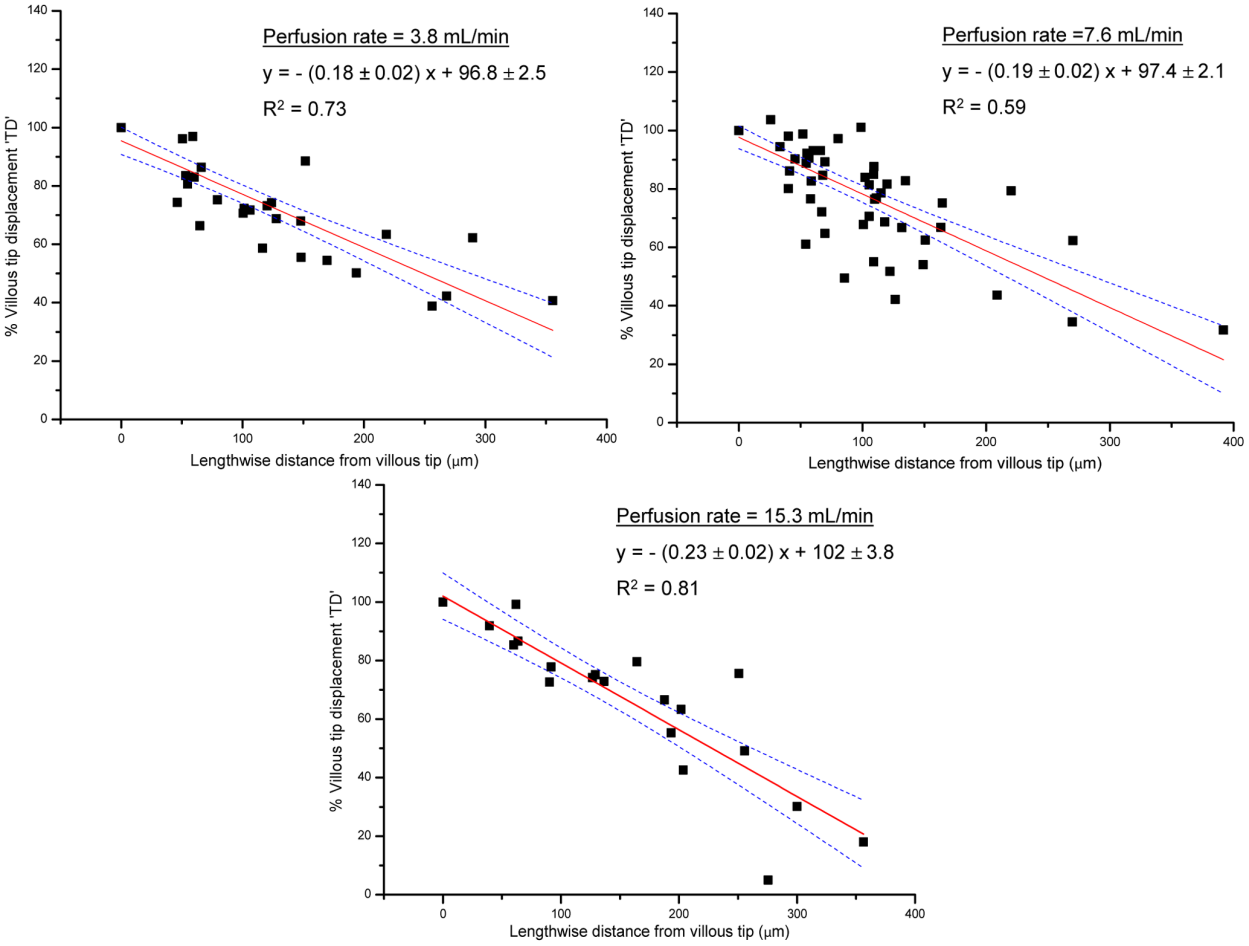
Relationship between displacement of points along the villous length with distance from the villous tip. The plot utilized data pooled from all villi*. Displacement is expressed as a percentage of total tip displacement (TD). The regression lines that best fitted the data obtained at each perfusion rate were all linear. * R^2^ values shown are for pooled data. R^2^ values for SLRs of individual villi were all above 0.8 at each flow rate. The dotted lines are 95% confidence intervals.

Curve fits of pooled data (CurveExpert v.1.4) from all flow rates showed that the variation of displacement (expressed as a percentage of TD) against distance down the villus was best fitted by a linear function (Fig. 7). At flow rates of 3.8, 7.6 and 15.3 mL/min significant linear regressions (Fig. 7) with R^2^ values of 0.73, 0.59, and 0.81 respectively were obtained. Moreover, the slopes of the linear fits at the three perfusion rates did not differ significantly on Student's t-test (d.f. = 29, T = 0.35, P>0.05 between 3.8 and 7.6 mL/min, d.f. = 24, T = 1.41, P>0.05 between 7.6 and 15.3 mL/min and d.f. = 13, T = 1.77, P>0.05 between 15.3 and 3.8 mL/min). Together these findings indicate that the villi were effectively rigid and did not bend at physiological or supra-physiological flow rates. Furthermore, the shafts of villi pivoted little around their bases. In regard to the latter finding it is important to note that the tension in the tightly mounted tissue (which was necessary to avoid movement of the entire mucosa on the probe) may have restricted such pivoting.

### Spatiotemporal maps along the longitudinal axis of villi

The patterns of distribution of strain rate about the longitudinal axis of three villi on spatiotemporal maps taken at all three LOIs showed no coherent patterns of phasic change in strain rate that would be consistent with local phasic contractile activity that affected the longitudinal dimensions of the villi.

## Discussion

This is the first study to assess the bending and passive mechanical properties of villi in the absence of any active villous motions in response to a flow of Newtonian fluid across living villous mucosa. It is noteworthy that the mucosa was harvested with minimal trauma and manipulation and was then maintained in a fluid environment similar to that *in vivo*. Hence whilst production of excess mucus by anoxic or mechanical degranulation of goblet cells was avoided, any material that is normally adherent or close to the villus [17], [29] was maintained *in situ*.

The lack of any significant change in the profiles of lines of defined points on villi even when villi were exposed to supra-physiologically high levels of shear stress, indicates they would behave as rigid structures within the gut. Hence villi would be sufficiently rigid to allow their shafts to tilt in concert with folding of the underlying mucosa to generate crowding of the villous tips between adjacent folds [6]. The ileal villi in this species were of similar lengths to those of other common laboratory animals such as rats (250–375 µm) [30], pigs (275–310 µm) [31] and dogs (460–550 µm) [32] and were within the range of villi lengths of the human small intestine (500–1000 µm) [33], [34]. Further, they were tubular of form.

The finding that villi were relatively rigid structures fits with the dynamics outlined by previous workers [35]. These workers concluded that villi would be in a turgid state as a result of absorbed fluid, the resultant interstitial pressure being opposed by tension of interstitial fibres and smooth muscle cells [10], [36] within and at the bases of these structures. Such turgor would render the villous shaft resistant to bending [35]. It may be considered that this turgor was augmented in our *ex vivo* preparation, in the absence of any ongoing venous or lymphatic drainage. However, the finding that TD did not decrease significantly with time counts against such a hypothesis as does the finding that the widths of individual villi did not change appreciably over the course of the experimental determination of their bending and velocity profiles. Hence it seems likely that ongoing activity of smooth muscle in the villous shaft [35] prevented such engorgement. Whilst the villi in our experiment did not exhibit spontaneous movement, the phasic contraction of myocytes associated with any such action would presumably augment intravillous pressure and further limit the capacity of the villus shaft to bend. The absence of spontaneous villous contractions in the *ex vivo* preparations is in line with the findings of previous workers [10] who reported that spontaneous longitudinal movements only occurred *in vivo*.

Although the shafts of villi were rigid, there was detectable movement of the villous tip during flow (<7% of the mean width of a villous). This movement was likely from pivoting of the shaft of the villus around the point of the attachment of its base to the mucosa and from relative movement of the point of attachment across the underlying muscular wall of the ileum. Given the likely restriction of translational and pivoting movements of villi from the taut mounting of the tissue on the probe, it is probable that these movements would be of greater magnitude *in vivo*. The presence of such movements supports the hypothesis that submucosal laxity allows mucosal folding and that villi between mucosal folds may incline toward each other enhancing villous crowding [6].

This is the first study to quantify flow patterns around living villi. Whilst much is known of the general nature of flow around rigid structures, notably the tendency for vortices to form between structures that project into a flow-stream [37], the effects of the material properties of the villi and of the mucins around and between the villi have not been previously described. The fact that the velocity of flow in the perivillous spaces around the bases of villi was reduced to levels that approximate those of macromolecular diffusion, even at supra-physiological rates of perfusion, fits with previous work indicating that the bulk of absorption occurs in the upper third of the villus [2], i.e. over a distance of 180 µm proximal to the tip of the villous. Any solvent drag [38] generated in the preparation, would similarly be around the villus tip and unlikely to influence mixing in the lower intervillous space. Thus convective mass transfer in these regions can only be secured by movements of the villi or mucosa [5]. Such mixing could be brought about by uncoordinated, i.e. spontaneous, contraction of villi [7], [8], [9], [10] or by mucosal folding [6].

While the size and form of the ileal villi of the possum are similar to those of humans, and mucosal architecture, i.e. density of villi and effective surface area per unit villus, are also similar over a range of laboratory species [39], caution is required in generalising the findings from this study. It is likely that flow dynamics vary with the rate and character of mucus secretion where the latter varies regionally along the small intestine [40]. Again it is possible that ileal villi of other vertebrate species vary in their rigidity, for example according to the abrasive qualities of the diet.

In conclusion, our experiments provide new insights into the ability of static villi to augment mass transfer and absorption. The lack of significant convective mixing in the base of the intervillous space indicates that absorption is likely to be confined to the tips of villi under conditions when the villi and wall are immobile. The finding that villi are resistant to bending, even at supra-physiological flow rates, indicates that intrinsic movements of villi will be correspondingly limited but that relative movements of their shafts will be reflected in movements of their tips. Correspondingly, the finding of displacement of the tips by flow indicates that the shaft of the villi may become angulated relative to the mucosa and supports a mechanism of fluid displacement by villous apical crowding and consequent augmentation of mixing [6].
